# Drivers of Tree Growth, Mortality and Harvest Preferences in Species-Rich Plantations for Smallholders and Communities in the Tropics

**DOI:** 10.1371/journal.pone.0164957

**Published:** 2016-10-20

**Authors:** Huong Nguyen, Jerome Vanclay, John Herbohn, Jennifer Firn

**Affiliations:** 1 Tropical Forests and People Research Centre, University of the Sunshine Coast, Sippy Downs, Sunshine Coast, Queensland, Australia; 2 School of Agriculture and Food Sciences, The University of Queensland, Brisbane, Queensland, Australia; 3 School of Environment, Science and Engineering, Southern Cross University, Lismore, New South Wales, Australia; 4 Faculty of Science and Technology, School of Earth, Environmental and Biological Sciences, Queensland University of Technology, Brisbane, Queensland, Australia; University of California Davis, UNITED STATES

## Abstract

There is growing interest in multi-species tropical plantations but little information exists to guide their design and silviculture. The Rainforestation Farming system is the oldest tropical polyculture planting system in the Philippines and provides a unique opportunity to understand the underlying processes affecting tree performance within diverse plantings. Data collected from 85 plots distributed across the 18 mixed-species plantations in the Philippines was used to identify the factors influencing growth, probability of harvest, and death of trees in these complex plantings. The 18 sites (aged from 6 to 11 years at time of first measurement) were measured on three occasions over a 6-year period. We used data from the first period of data collection to develop models predicting harvesting probability and growth of trees in the second period. We found little evidence that tree species diversity had an effect on tree growth and tree loss at the community level, although a negative effect was found on tree growth of specific species such as *Parashorea plicata* and *Swietenia macrophylla*. While tree density of stands at age 10+ years (more than 1000 trees/ha with diameter > 5cm) did not have an impact on growth, growth rates were decreasing in stands with a high basal area. Tree size in the first period of measure was a good predictor for both tree growth and tree status in the next period, with larger trees tending to grow faster and having a greater chance of being harvested, and a lower possibility of mortality than smaller trees. Shade-intolerant trees were both more likely to be harvested, and had a higher probability of death, than shade-tolerant individuals. Native species and exotic species were equally likely to have been lost from the plots between measurement periods. However, shade-tolerant native trees were likely to grow faster than the others at age 10+ years. Our findings suggest that species traits (e.g. shade tolerance) could play an important role in optimizing species composition for this type of plantation. Shade-intolerant species with rapid early growth could contribute early income for farmers in mixed plantings where some products may take years to realize. We also suggest selective harvesting or thinning (for small shade-intolerant trees) applied at age 10+ years could reduce the competition for resources between individuals.

## Introduction

In the tropics there is increasing interest in establishing mixed species plantations for a wide range of economic, silvicultural and sustainability objectives [1–3](). This is in contrast to dominance of monocultures in ‘industrial’ plantation development in the tropics and temperate regions, largely because of the associated economic benefits. Smallholder and community forestry are abundant in the tropics [4] and several countries have created national reforestation programs aimed at encouraging landholders to plant mixtures, e.g. Vietnam, China and the Philippines [5]; especially involving combinations of native species for which there is often little comprehensive information. In certain situations, mixed species plantations are found to be more successful than monocultures in biomass production and carbon sequestration [6, 7], improved nutrient cycling [8, 9], reduced damage from pest or disease [10, 11], and improved financial benefits by diversifying products [3].

Mixtures can provide financial and livelihood benefits that are attractive to smallholders and local communities because they may provide a more diverse range of products, such as fruit, crop, resin, early timbers of short-lived species or valuable timbers of long-lived species [12–14], which may help farmers diversify their subsistence and capital investment. Mixtures can also generate financial returns in both the short-term (harvesting opportunities of faster-growing species) and in over the longer term (harvesting opportunities of slower-growing but potentially more valuable species). The diversity of species with differing traits also means mixtures can provide localities with more resilient forests in the face of ecological disturbance and climate change impacts [15] and may be better able to continue to provide income to smallholders despite fluctuations in markets [16]. As potential financial returns from mixtures are recognized more widely, many farmers and local communities seek to use mixed species plantations, often with native species for their small-scale plantings [17–20].

Although mixed-species plantations can have many benefits for smallholders and communities, none of these benefits are assured [3] because of substantial knowledge gaps concerning the combination and performance of species in these complex plantings remain [7, 17, 21]. In addition, most established plantations using native species are still young and have yet to reveal their overall performance [3, 17]. Apart from social challenges [11], the primary technical difficulties in using mixed-species plantations are the choice of species to use in the mixture and the silvicultural techniques needed to achieve high productivity [2]. Interactions (facilitation or competition) that occur in mixtures drive the growth of individual species, and the relative influence of these interactions is likely to change stand development [17, 19, 22]. When the choice of species used at particular sites is inappropriate one species may suppress the growth of the others, and mixtures may be less productive [2, 23, 24]. Therefore, mixtures are often restricted to relatively small areas or to situations where diversifying production is a great advantage, such as for small farmers with limited resources [25].

Understanding of tree mortality is central to any prediction of forest dynamics because the long-term dynamics of woody biomass are regulated by the difference between gains through individual growth and losses through mortality [26]. The growth and mortality of forest trees is dependent on impacts of various factors such as species identity, tree vigour and size, and environmental factors on the interactions and processes in stands [26–28]. While differences in mortality rates among species appear to be major determinants of ecological succession [29] and stand structure [30, 31], tree vigour and size are known as possible indicators of performance of a species [32–35] because size partially reflects competitive ability of a tree [36]. Plant functional traits have been also found to predict relationship with growth-mortality trade-offs [35, 37–41].

The Rainforestation Farming system in the Philippines (called the rainforestation plantings hereafter) was one of the oldest tropical polyculture planting systems using a mix of native species, along with exotic species, for both socio-economic and ecological purposes [42, 43]. In the Philippines, understanding of silvicultural requirements and growth rates of species in the Dipterocarpaceae family (that are regarded locally as having highly valued timbers) and other native tree species is limited [44, 45] and knowledge about habitat ranges of many native species is lacking (e.g. species-site matching, site requirements for the long-term sustainability of reforestation efforts) [44, 46].

In this study, we investigate the factors influencing growth, probability of harvest, and death of trees to assist understanding of the underlying processes affecting tree performance within diverse plantings to answer the question that if the design of the rainforestation plantings is suitable for smallholders and communities. To achieve this, we investigate species performance and species utilization in these plantations by addressing the following three hypotheses:

1. Species attributes are often used as possible indicators of the performance of species grown in a mixed stand [47, 48]. From this view, we hypothesize that species with different origin (i.e. native or exotic) and shade tolerance (i.e. shade-intolerant or shade-tolerant) planted in the rainforestation plantations may exhibit different growth and mortality. This may in turn influence harvesting preferences of farmers in terms of species and the timing of harvest.
2. Patterns of tree growth, mortality and harvest may be driven by tree size, species diversity, tree density and stand productivity (i.e. stand basal area) because these indices are surrogate measures for competition in a mixed species planting.
3. The patterns of tree growth, mortality and harvest might also be affected by the geographical characteristics of planting site. All the tested factors are easily measured and accessible indices in the field.

Finally, we apply our results to make recommendations for species selection, design and silvicultural practices of mixed species plantations for forestry programs and landowners in tropical regions.

## Materials and Methods

### Ethics Statement

The study was a part of two smallholder projects funded by the Australian Centre for International Agricultural Research (ASEM/2006/091 and ASEM/2010/050). These projects belonged to the Australian Aid Program that encourages Australia’s scientists to use their skills for the benefits of developing countries and Australia. The study was conducted in Leyte province, the Philippines and with the permission of the owners of the plantations studied (for more details see Nguyen et al. [49]) and we obtained all relevant permissions from the relevant Filipino agencies to conduct this research. It was not possible to sample from all 28 sites that were established under the Rainforestation Farming system because several plantations had been detrimentally affected by fire, harvesting, clearing for other agricultural activities; because access was not granted by the land owners; or did not meet minimum requirements for measurements (e.g. trees greater than 5 cm diameter).

### Study Area

The study was conducted in Leyte province, in the eastern Philippines. Leyte Province has a humid monsoon climate and the average rainfall in the study area for the years 1980–2000 was 2,686 mm with an annual variation of between 1,775 mm in 1987 and 3,697 mm in 1999 [50]. Although there is no pronounced dry season, the region experiences its lowest rainfall of less than 100 mm per month between March and May [51]. Dry periods of several months duration with rainfall of less than 100 mm per month can sometimes occur, as was the case during the ‘El Nino’ year of 1993, the year in which the project commenced. The average annual temperature is 27.5°C and ranges from 26.3 to 28.7°C. The relative humidity is always high and the average monthly level for the years 1980–2000 ranged from 75.1% in March to 80.1% in October [50]. The soils are derived from volcanic parent material and were slightly acidic with a pH 4.1–4.9 [52].

All study site were small-scale mixed-species plantations arising from the Rainforestation Farming program. This program was initiated in 1992 and resulted in the establishment of 28 plantations on land owned by local communities and private properties in Leyte province in the Philippines [43]. Approximately 100 species were used in the program, including endemic pioneer tree species, longer-lived species, mostly of Dipterocarpaceae, fruit trees and a limited number of exotic timber species. Various combinations of these were used to create a series of small-scale (about one hectare) plantations on private land [43]. At the beginning of the project, pioneer and light demanding species were planted at a spacing of 2 x 2 m, and in the second year, shade-tolerant, as assessed by local knowledge, timber and fruit tree species were inter-planted at a general spacing of 2 x 1 m [42].

### Data Collection

Data were collected from 85 permanent plots distributed across the 18 mixed-species plantations belonging to the Rainforestation farming system [49, 53–55]. The first measurement of species composition and stand structural characteristics was undertaken in 2006 when the trees were aged between six and eleven years. Measurements of trees and site properties were collected from the circular plots with a radius of 5 m (78 m^2^ area) randomly located within the plantations [4]. The number of plots sampled at each farm ranged from 1 to 12 plots depending on the size of the farm’s plantings, with the number being determined by the size of site so that the sampling area occupied at least 5% of site [56, 57]. All plots and trees within them were permanently marked in the field. In each plot, all trees were counted and identified for species and origin (i.e. native or exotic) and the diameter at breast height was measured. Each plot contained at least seven trees greater than 5 cm in diameter. All the plots were re-measured in 2008 and again in 2012, at which time the DBH was measured along with information whether the tree was still present (i.e. survival), and if not, whether it had died of natural causes or had been harvested for the period of 2006–2008 or 2008–2012. A distinction was made between harvesting and natural tree deaths based on the evidence in the form of stumps or dead stems. Site/plot characteristics such as soil type, slope, elevation, and location of plots (i.e. at edge or center of planting) were recorded (Table 1). The shade tolerance of these species was described by Schulte [42].

**Table 1:** Variables used in the modelling.

Variable	Description	Unit	Mean value (Range)
Dependent variables			
Tree growth (PAIBA)	Average annual growth in cross-sectional area of a tree between two consecutive measurements	cm^2^/year	23.60 (0.001–271.44)
Tree status (S)	Likelihood of a tree in being alive (A), harvested (H) or dead (D)		
Explanatory variables			
*Tree and species characteristics*:			
DBH	Diameter (over bark) at the breast height (1.3 m)	cm	13.8 (5.0–49.3)
Origin	Origin of species planted: exotic or native		
Shade	Shade tolerance of species: SI = shade-intolerant or ST = shade-tolerant		
*Stand characteristics*:			
Species richness	Number of species at each plot		5 (2–14)
Effective species richness (e^H^)	Effective species richness at each plot; H = Shanon’s index		4.4 (1.4–11.9)
Tree density at period start (2006)	Number of trees per ha measured at each plot in 2006	trees/ha	1645 (764–6621)
Tree density at period end (2012)	Number of trees per ha measured at each plot in 2012	trees/ha	1309 (0–5475)
Stand basal area (BA)	Total basal area of all trees in each plot counted per ha	m^2^/ha	24.9 (5.1–75.5)
*Plot and site characteristics*:			
Slope	Degree slope measured looking down-slope on a six-point scale: 1 = flat 0–3°; 2 = gentle 4–8°; 3 = moderate 9–16°; 4 = steep 17–26°; 5 = very steep 27–45°; and 6 = precipitous > 45°		3 (1–6)
Location	Location of plot in site (i.e. center or edge)		
Soil type	Limestone or volcanic		

Explanatory variable ‘species richness’ was excluded from modelling because its high VIF (19.42) and high correlation with ‘e^H^’ (correlation = 0.96).

Tree basal area was derived from the DBH of each measured tree in the plots. Periodic annual increment of tree basal area (PAIBA) was calculated as the average annual tree growth between two consecutive measurements. Mean DBH and mean PAIBA of species were calculated across all the individual trees of each species (S1 Table). Tree species diversity was measured by species richness (i.e., number of species observed) and effective species diversity (i.e. exponential Shannon’s index that takes into account both species diversity and evenness within stand) on each plot. The proportions of survival trees, harvested trees and dead trees were calculated for each species.

The statistical analysis was conducted over two periods of measurement, 2006–2008 and 2008–2012.

### Data Analysis

**Fig 1:**
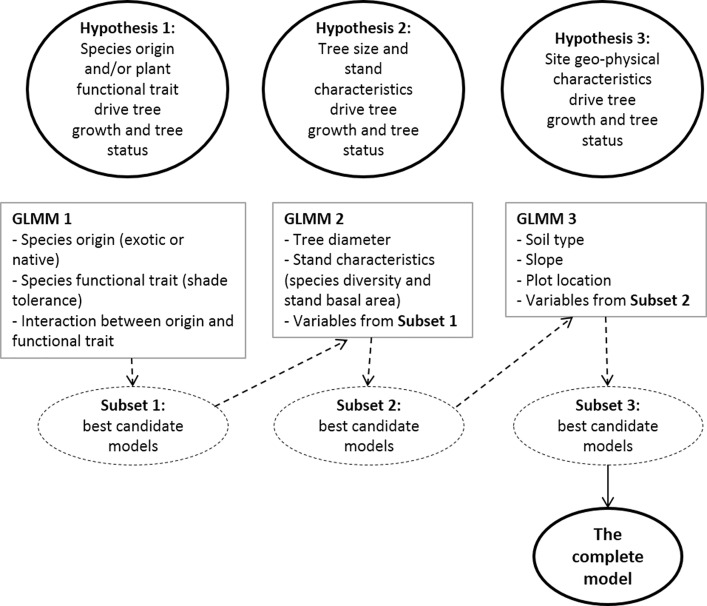
Schematization of model construction for tree growth and tree status based on characteristics of tree, species, stand and site.

We used Generalized Linear Mixed-Effects Models (GLMMs) [58] to test the hypotheses and the package ‘lmerTest’ to evaluate model fit and significance of random and fixed effects [59]. All analyses were conducted in R 3.1.0 [60]. Three global GLMMs were built in order to examine each outcome, i.e. responses of tree growth or tree status (i.e. survival, dead or harvested, in the relationship with characteristics of tree/species (i.e. DBH, species shade tolerance and species origin), stand structure (i.e. species richness indices, tree density and basal area of stand) and/or site description (i.e. plot location, slope and soil type) (Table 1) as explanatory variables, and a random effect of plot nested in site. The indices measured in the previous period (2006–2008) were used as explanatory variables to predict tree growth and tree status in the subsequent period (2008–2012). Models were developed to identify factors influencing variation in the performance of individual trees in community (called community models hereafter). These models used the combined data from the 32 common species that comprised 96.3% the data and species models were applied for some individual species. The scheme of model construction was presented in more detail in Fig 1.

Prior to developing multiple regression models, all explanatory variables were tested for multi-collinearity by calculating the variance inflation factor (VIF) of variables using package ‘*usdm*’ (S2 Table). Variables with VIF < 3 (low multi-collinearity) were considered for inclusion in the models whereas variables with VIF > 3 were examined and eliminated if it was theoretically sound, that is, measuring conceptually similar things [61]. Global model GLMM1 included variables of species origin and shade-tolerance to examine if species origin, species shade-tolerance or origin–shade-tolerance interaction drove the tree growth or probability of trees surviving or disappearing from the plot (through death or harvesting). A full set of candidate models was generated from all combinations of these fixed effect variables. The multimodel inference method in package ‘*MuMin*’ using model averaging was applied to select the best candidate models based on the REML criterion (residual maximum likelihood) [62]. Weight of candidate models and relative variable importance (RVI) of variables were calculated in the process based on AICc (the second order Akaike Information Criterion) of candidate models [63]. Subset 1 of best candidate models selected from GLMM1 included the best model with the lowest AICc and other supporting models with ΔAICc < 2 [64] (S3 and S4 Tables). Then the variables from subset 1 were combined into global model GLMM2 to test for their effects when controlled by other variables from hypothesis 2. The statistical process was repeated for GLMM2, and then global model GLMM3 to select the best predictors for tree growth and tree status in the stands. Finally, subset 3 of best candidate models selected from GLMM3 contained the most important explanatory variables to be used to build the complete model that synthesized all the three hypotheses (Fig 1, S3 and S4 Tables). This complete model tested effect of each variable in the complex relationship with other potential variables. Wald statistics were used to test significance of fixed variables in the final models.

For the model of tree status, regression coefficients of variables from the final GLMM model were extracted for each output category (dead or harvested) by a multinomial logistic regression model, using package ‘*nnet*’. Equations predicting the probability for each output category (dead or harvested) took the form *ln(P) = α*_*0*_ *+ α*_*1*_*x*_*1*_ *+ α*_*2*_*x*_*2*_ *+ … + α*_*n*_*x*_*n*_ (where P is probability, *α*_*0*_, … *α*_*n*_ are regression coefficients of explanatory variables *x*_*1*_, … *x*_*n*_). Category ‘survival” was set as reference in the modelling.

Then, hypotheses 2 and 3 were also tested for individual-species using the same statistical method to identify factors influencing variation in the performance of individual trees of specific species. The growth models of individual-species were built for each of the 14 most common species with at least 20 trees measured for each species; whereas the status models for only 7 common species with at least 20 trees measured and 5 lost trees recorded for each species, including *Melia dubia*, *Gmelina arborea*, *Terminalia macrocarpa*, *Swietenia macrophylla*, *Vitex parviflora*, *Gymnostoma rumphianum* and *Pterocarpus indicus*.

## Results

### Overall growth, mortality and harvest of common species

**Fig 2:**
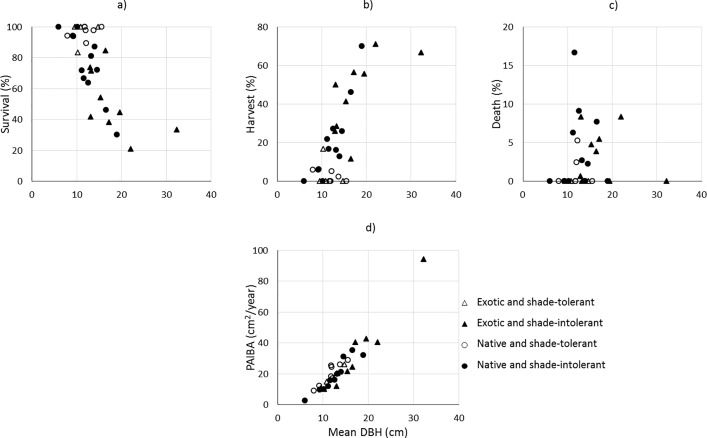
Distribution of the likelihood of tree status and tree growth along with mean DBH of the common species in the Rainforestation plantings. a) proportion of survival trees; b) proportion of harvested trees; c) proportion of dead trees; and d) tree growth rate.

We found 32 common species amongst the Rainforestation plantations including 4 shade-tolerant exotic species, 9 shade-intolerant exotic species, 8 shade-tolerant native species and 11 shade-intolerant native species (S1 Table), and comprised of 96% total trees measured in the Rainforestation plantings. Differences were found for the likelihood of tree status (survival, death and harvest) and tree growth at these stands (Fig 2). A higher survival rate was observed with shade-tolerant species (e.g. *Parashorea plicata* and *Shorea palosapis*); whereas, shade-intolerant species were more likely to be harvested (e.g. *Swietenia macrophylla*, *Gmelina arborea*, *Gymnostoma rumphianum*, *Terminalia macrocarpa* and *Vitex parviflora*). The mortality rate was likely low in shade-tolerant species (less than 5% over the 6 year period). As we expected, species with a larger mean DBH (e.g. *Leucaena leucocephala*, *Melia dubia*, *Gmelina arborea* and *Samanea saman*) tended to have a faster-growth rate, a low survival rate and a higher probability of being harvested than species with smaller mean DBH.

### Predicting growth and status of tree at community level

*Tree growth rate*: The growth rate of individual trees differed widely in the mixed plantations, ranging from 0.001 to 271.44 cm^2^/year. Some species, e.g. *Melia dubia*, *Gmelina arborea* and *Terminalia macrocarpa* had higher variation in growth rate than the others.

**Fig 3:**
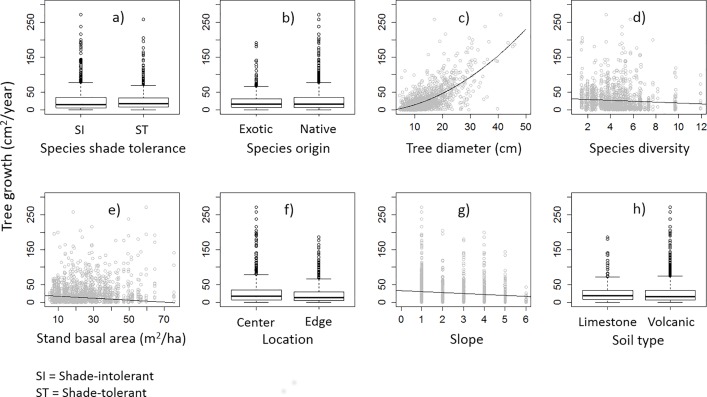
Characteristics of tree, species, and stand predicting the growth rate of trees in the Rainforestation plantings. a) Species shade torance; b) Species origin; c) Tree diameter; d) Species diversity; e) Stand basal area; f) Location; g) Slope; and h) Soil type.

Tree and species characteristics (i.e. DBH, origin and shade), stand structure (i.e. e^H^ and BA), and plot and site characteristics (i.e. location, slope, soil type) had high relative importance in predicting the variance in tree growth over the two time periods (Table 2), with the exception being tree density. This suggests that tree growth in the stands was not influenced by tree density from the time period before. The final growth model showed that growth of individual trees in the community was predicted by tree DBH in the first time period, species origin (whether native or exotic) or the interaction between origin and shade-tolerance (Table 2, Fig 3). For example, tree DBH was positively correlated to growth rate; whereas, native species was negatively correlated. This result suggests larger trees were still growing well in the subsequent period; yet individuals of native species grew more slowly than exotic species. Shade-tolerant and -intolerant species surprisingly did not show significant differences in growth rates although shade-tolerant species tended to grow more slowly than shade-intolerant species. However, individuals of shade-tolerant native species tended to grow faster than the other individual trees. A significant negative relationship between tree growth and stand basal area suggests that trees in stands with higher basal area grew slower than in stands with lower basal area in the later period. Our results also showed that tree growth depended on site-level factors such as slope and soil type; suggesting that trees in these stands might grow faster on low slope and on volcanic soil (Table 2, Fig 3). Other factors including species shade-tolerance, species diversity and plot location, although important in the model, were not found significant to tree growth in these stands.

**Table 2:** Results from the final linear mixed-effects models predicting growth and status of trees (dead or harvested) in the community of 32 common species. RVI = Relative variable importance.

Growth model		Status model		
*RVI of variables*		*RVI of variables*		
Shade	1.00	Shade	1	
Origin	1.00	DBH	0.88	
Shade x Origin	1.00			
DBH	1.00			
e^H^	0.54			
BA	0.96			
Location	0.76			
Slope	0.82			
Soil type	0.94			
*Fixed effects*		*Fixed effects*	Dead	Harvested
(Intercept)	-2.91	(Intercept)	-2.92***	-2.64***
Shade(tolerant)	-4.38	Shade(tolerant)	-1.88***	-2.76***
Origin(native)	-4.14*	DBH	-0.08***	0.05***
Shade(tolerant) x Origin(native)	13.49**			
DBH	1.36***			
(DBH)^2^	0.07***			
e^H^	0.54			
BA	-0.28***			
Location(edge)	-2.06			
Slope	-1.93*			
Soil type(volcanic)	7.88*			
*Random effect (%)*		*Random effect (%)*		
Site	9.2	Site	2.8	
Site (Plot)	7.7	Site (Plot)	9.6	
Residual	83.1	Residual	87.6	
*Correlation*		*Correlation*		
R^2^(fixed effects)	0.49	R^2^(fixed effects)	0.03	
R^2^(fixed + random effects)	0.58	R^2^(fixed + random effects)	0.15	

Significance levels

† p < 0.1

* p < 0.05

** p < 0.01, and

*** p < 0.001.

Categorical variables ‘shade (intolerant)’, ‘origin (native)’, ‘location (center)’, ‘soil type (limestone)’ and ‘status (alive)’ were set as reference in the modelling.

The variation in tree growth was mostly explained by 49% of fixed effects (R^2^(fixed effects) = 0.49) and only 9% of random effects (R^2^(random effects) = 0.09). This indicates that the variation was larger between individual trees rather than between sites or plots (Table 2).

**Fig 4:**
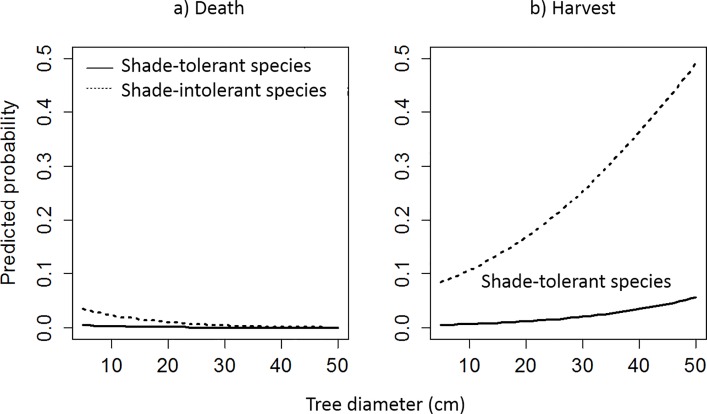
Probability of a tree in the likelihood of death or harvest along tree size (DBH) of different functional groups (shade-tolerant and shade-intolerant species) in the Rainforestation plantings. a) death and b) harvest.

*Tree status*: *being harvested or dead*.- Variables associated with stand structure or geographical characteristics were not important to the status of a tree (i.e. being dead or harvested), suggesting that species diversity, tree density, or stand productivity did not drive the status of trees in these plantings. The important variables including tree diameter and shade-tolerance of species were significant predictors of the status of a tree (Table 2). Larger trees in the stands were predicted to have a higher probability of being harvested in the subsequent period (Fig 4A and 4B) whereas smaller trees had a higher probability of mortality. A higher probability of harvesting or mortality was found for shade-intolerant trees in the later period. Conversely, shade-tolerant trees were predicted to have low mortality and only a small likelihood of harvesting in the next period (Fig 4A and 4B).

The equations for the probability of harvesting or death of a tree in the stands were presented below and in Fig 4A and 4B.

ln(PdeadPsurvival)=−2.917−1.897×Shadetolerant−0.079×DBH

and

ln(PharvestedPsurvival)=−2.640−2.761×Shadetolerant+0.052×DBH

where *P*_*survival*_ and category “shade-intolerant” were set as reference in the model.

These equations indicated that probability in the likelihood of death of shade-tolerant trees was 0.15 times (= *e*^-1.879^) lower than that of shade-intolerant trees; whereas probability in harvesting of shade-tolerant trees was only 0.06 times (= *e*^-2.761^) lower than shade-intolerant trees. The probability in death decreased 0.92 times (= *e*^-0.079^) and the probability in harvesting increased 1.05 (= *e*^0.052^) times when tree diameter increased 1 cm respectively.

Random effects in the tree status model indicated that variation in tree status was mostly between individual trees rather than between plots or sites (Table 2).

### Predicting growth and status of tree at population level

*Tree growth*.- We found that explanatory variables at tree or stand level were important to the growth of the 14 common species; although each species was affected by different combinations of variables (Table 3). Similar to the results from community models, tree size (DBH) had a significant effect on tree growth of six species, with the larger trees of these species growing well in the later period. Species diversity had significant negative effect on tree growth of *Parashorea plicata*, indicating that growth of this species was slower in species-rich stands. Stand basal area also had significant negative effect on tree growth of some species (i.e. *Parashorea plicata*, *Swietenia macrophylla*, *Shorea contorta*, and *Hopea plagata*), suggesting that growth of these trees tended to decrease within highly productive stands. *Terminalia macrocarpa* and *Gymnostoma rumphianum* trees were likely to grow slower on steep slopes as a significant negative relationship was found between slope and tree growth of these species. Only individual trees of *Swietenia macrophylla* planted on volcanic soil grew significantly better than on limestone soil in the later period, suggesting this species is suitable on volcanic rather on limestone soil. Soil type was found not significant to tree growth of the other species. Individuals of *Parashorea plicata* (a shade-tolerant Dipterocarp species) planted at the edge of site grew slower than those at site center; whereas *Gymnostoma rumphianum* individuals (a shade-intolerant species) grew better at site edge because of receiving more light.

**Table 3:** The final linear mixed-effects models predicting tree growth of individual species. RVI = Relative variable importance.

	*Melia dubia*	*Parashorea plicata*	*Dracontamelon dao*	*Gmelina arborea*	*Terminalia microcarpa*	*Swietenia macrophylla*	*Shorea palosapis*	*Vitex parviflora*	*Gymnostoma rumphianum*	*Pterocarpus indicus*	*Shorea polysperma*	*Tectona grandis*	*Shorea contorta*	*Hopea plagata*
*RVI of variables*														
DBH	0.87	1.00	1.00	0.93	1.00	1.00	1.00	1.00	1.00	1.00	1.00	1.00	1.00	1.00
e^H^	0.58	0.95	0.88	0.58	0.67	0.88	0.85	0.59	0.65	0.68	0.91	0.83	0.86	0.45
BA		1.00					0.48				0.63	0.30	0.95	0.42
Location	0.95	0.98	0.95	0.98	0.89	0.74	0.94	0.80	0.99	0.74	0.96	0.73	0.87	0.88
Slope	0.75	0.67	0.94	0.71	0.97	0.66		0.73	1.00	0.58	0.83	0.86	0.83	0.43
Soil type	0.89		0.95	0.96	0.92	1.00		0.95	0.89	0.71				
*Fixed effect*														
(Intercept)	69.45	58.02**	27.96	14.02	21.28	-3.95	21.86	39.70	-1.44	-17.85	-20.04	-59.71	-17.82	4.68
DBH	-5.25	-0.02	1.82	0.42	-0.18	0.31	0.51	-5.45**	3.52*	1.59	1.40	2.90	4.23**	0.35
(DBH)^2^	0.17	0.21***	0.01	0.03	0.15***	0.11***	0.13†	0.30***	-0.03	0.03	0.11	0.01	0.03	0.16
e^H^	-0.88	-5.79**	4.10†	0.19	-2.20	1.66†	-4.52†	-0.63	-1.57	1.93	9.40	2.18	3.65	-0.49
BA		-1.22***				-0.21*	-0.62†				-0.79	0.26	-1.03**	-0.25**
Location (edge)	37.94	-14.50**	-12.24	33.32	-6.10	-0.86	9.56	0.28	15.91*	2.39	-23.95	1.51	0.42	-4.10
Slope	-5.73	-1.24	-11.24	2.15	-6.76*	-0.81		-5.11	-8.64***	-1.30	-1.72	6.78	1.40	0.82
Soil type (volcanic)	-13.25		5.94	-25.10	0.01	8.20*		6.81	4.12	1.12				
*Random effect (%)*														
Site (Plot)	74.8	17.8	0.0	27.4	10.5	21.6	11.2	29.7	3.8	8.7	54.9	0.0	38.1	0.0
Site	0.0	7.2	83.7	47.5	7.2	0.0		25.8	0.0	0.0	0.0	0.0	44.5	0.0
Residual	25.2	75.0	16.3	25.1	82.3	78.4	88.8	44.5	96.2	91.3	45.1	100.0	17.4	100.0
R^2^(fixed effects)	0.26	0.72	0.24	0.42	0.72	0.67	0.71	0.33	0.68	0.62	0.34	0.60	0.54	0.61
R^2^(fixed+random effects)	0.86	0.80	0.88	0.86	0.76	0.73	0.77	0.57	0.77	0.64	0.71	0.60	0.92	0.61
AICc	227	1151	509	325	1359	1905	344	1118	615	378	742	365	840	476

Significance levels

† p < 0.1

* p < 0.05

** p < 0.01, and

*** p < 0.001.

Bold numbers refer to significant coefficients in the models.

Categorical variables ‘location (center)’, ‘soil type (limestone)’ were set as reference in the modelling.

Random effects in the models indicated that variation in tree growth was higher between trees than between plots or sites for most of these 14 species; the variation, however, was high between plots or sites for two species (i.e. *Melia dubia* and *Dracontamelon dao*).

*Tree status*.- The species models showed only stand basal area (BA) and soil type were important to the status (dead or harvested) of a tree for five species *Melia dubia*, *Gmelina arborea*, *Terminalia macrocarpa*, *Vitex parviflora* and *Gymnostoma rumphianum* (Table 4); none of explanatory variables found was important in the models of *Melia dubia*, *Swietenia macrophylla* and *Pterocarpus indicus*. However, BA was significant predictor for tree status of only one species i.e. *Terminalia macrocarpa*, showing that the probability in being harvested of this species increased dramatically but that in being dead decreased slightly for larger trees. Soil type influenced significantly tree status of only species *Gmelina arborea*, showing this species might have higher risk in mortality and lower chance of harvesting on volcanic soil. The variation of tree status was found to be higher between trees rather than between plots or sites for this species.

**Table 4:** Final linear mixed-effects models of tree status (dead or harvested) for individual species. RVI = Relative variable importance.

	*Melia dubia*		*Gmelina arborea*		*Swietenia macrophylla*		*Terminalia microcarpa*		*Vitex parviflora*		*Gymnostoma rumphianum*		*Pterocarpus indicus*	
*RVI of variables*														
BA							0.94							
Soil type	0.34		0.62				0.63		0.30		0.42			
*Fixed effect*	Dead	Harvested	Dead	Harvested	Dead	Harvested	Dead	Harvested	Dead	Harvested	Dead	Harvested	Dead	Harvested
Intercept	-9.61***	-0.12***	-10.373***	0.692***	-5.46***	-2.03***	-5.95	-12.79	-8.60*	-7.91*	-10.80	-12.80	-3.22	-2.12
BA							-0.07***	0.06***						
Soil type (volcanic)	7.53	-1.05	7.329*	-2.638*			3.16†	8.80†	4.43	5.45	7.56	11.55		
*Random effect*														
Site	0		30.2		0.3		6.4		0.0		0.3		0.0	
Site (Plot)	0		0.0		9.5		14.8		22.3		36.3		0.0	
Residual	100		69.8		90.1		78.7		77.7		63.4		100.0	
R^2^(fixed effects)	0.05		0.15		0.00		0.19		0.002		0.01		<0.01	
R^2^(fixed+random effects)	0.05		0.40		0.10		0.36		0.225		0.37		<0.01	
AICc	120		131		524		351		235		248		121	

Significance levels

† p < 0.1

* p < 0.05

** p < 0.01, and

*** p < 0.001.

Bold numbers refer to significant coefficients in the models.

Categorical variables ‘status (alive) and ‘soil type (limestone)’ were set as reference in the modelling.

## Discussion

Not surprisingly in a complicated tropical forest mixture like the Rainforestation plantings, we found tree growth and loss was explained by a combination of anthropogenic and natural factors, most notably tree/species and site characteristics and farmers’ harvesting preference. Variations in species composition and resource availability appear to have influenced the growth, survival, mortality and harvesting of trees of different species. Recognising the factors influencing growth and mortality of individuals of particular tree species during development of mixtures is important in species selection, design and silviculture of highly diverse plantings.

Previous studies have examined dynamics and outcomes of species-rich plantations, including a number of studies on biodiversity-productivity relationship, role of wood density, or growth and leaf trait of selected species within Rainforestation plantings [29, 49, 53, 54, 65]. Our study, however, is the first to investigate potential predictors of the performance of individual trees or species within highly diverse mixtures. We synthesize various impacts at tree, species and stand levels on growth and status of individuals for specific species in polycultures to provide insights into farmer’s preferences for harvesting of trees from polycultures.

The growth and status of individual trees in the following monitoring period (i.e. continued growth, death or harvesting) were found to be related to the characteristics of tree, species and stand. There was no evidence of impacts of overall species diversity in the tree community, yet a significantly negative effect was found for some species (i.e. *Parashorea plicata*) as a consequence of the high species diversity in the Rainforestation plantings during the study period.

Although the Rainforestation plantings had very high initial planting densities of approximately 5000 trees per ha, the residual density was typically around 1000 trees/ha of trees > 5 cm diameter at age 11–17 years because of high mortality in the early years [49, 54]. Tree density had no effect on tree growth or tree loss in this period; stand basal area (average 25 m^2^/ha at the Rainforestation plantings), however, had a negative impact on tree growth in the tree community possibly. da Cunha et al [66] found similar results in the Amazon forest that productivity starts to decline at a basal area of about 20–25 m^2^/ha and significantly falls off at > 25 m^2^/ha. Also, in the practical guidelines by Wadsworth et al. [67] a stand density of approximately 25 m^2^/ha is suggested. This trend likely occurred because as stand basal area increased competition for resources between trees increased, and therefore overall growth rate overtime even in the most productive stands was reduced.

The high productivity of stands might be consequence of a high stem density within the stands or alternatively because of some fast-growing trees. The former type of stand was also found in a similar system—ecological restoration plantations in the tropical North Queensland using more than 40 species in each plantation [68]. Stands that produced more biomass were often species-rich and stem-dense, yet most of stems grew more slowly and were in smaller size classes [68]. The apparent trend for tree growth and high stand basal area to be negatively related could reduce the timber production potential of stands. In this case, it is likely that thinning practices are needed for dense stands to reduce competition between individuals and improve growth of the stands [66, 67]; whereas more selective harvesting at stands comprising of some large and fast-growing trees might to leave more space for residual small trees.

Prior to the implementation of the Rainforestation plantings, farmers expressed a preference for exotic species, most likely due to perceptions of faster growth and a related ability to harvest them sooner compared to native species [43]. As expected, we found that exotic species had a significantly higher growth rate compared to native species. However, no difference in harvesting or mortality of individuals was found between these species groups in this period. This suggests that mortality probability and harvesting preference might be influenced by other factors rather than species origin. Shade tolerance of species did appear to explain both growth and the probability of being harvested and mortality. Shade-tolerant individuals had a lower probability of harvesting and survival over the period of measurement whereas individuals of native and shade-tolerant species tended to be fast-growing trees (e.g. Dipterocarpaceae species *Parashorea plicata*, *Shorea palosapis*, *Shorea polysperma*, and *Shorea contorta*).

The early harvesting of larger trees of fast-growing and shade-intolerant species at age 10+ would have reduced competition for resources and probably facilitated the growth of shade tolerant species remaining in the stands, and thus probably explains the higher growth rate of the group of native shade-tolerant trees. The higher rate of mortality in shade-intolerant species may involve changing light availability in plantings over time. This pattern is particularly strong in smaller statured shade-intolerant species [69]. Additionally, as stands age shade-tolerant species are possibly outcompeting pioneer and shade-intolerant species for resources [66]. After stands develop with time, small trees of pioneer and shade-intolerant species might be shaded by overstorey trees, resulting in slower growth and self-thinning. In conjunction with this, harvesting of pioneer and shade-intolerant trees of different sizes occurred, e.g. large stems being harvested for timber and small stems for firewood, although larger trees were preferred in harvesting than small trees at these stands. Shade-tolerant trees were less likely to be harvested because most of shade-tolerant species (e.g. often species from Dipterocarpaceae in the Rainforestation plantings) were slow-growing trees and were not target for the early harvesting by farmers; therefore, the importance of shade-tolerant species increased in the system as consequence of the loss of shade-intolerant trees [49].

Our study found that small individuals of shade-intolerant species in the plantings were likely to have higher risk of mortality because these trees were shaded by larger trees. This consequence driven by competitive thinning is often observed in young stands as the trees within these stands begin to show different rates of growth and resources (e.g. light become limiting with canopy closure) [70, 71]. For plants that are competing for light, shorter trees produce many of their leaves in the shade of taller neighbours, resulting in slow growth and in some case can result in death depending on the silviculture of the respective species [72, 73]. Such asymmetric competition for light provides the simplest explanation for greater mortality among small trees in stands [72]. The reason that the large trees still grew fast in the next period might be related to the stature-dependent shifts in allometry and photosynthetic capacity.

Finally, the Rainforestation plantings appear to have developed some typical characteristics of natural forests in dynamics of growth and mortality; shade-intolerant species and small trees had a higher mortality rate in the Rainforestation plantings. For instance in a natural forest in Borneo, higher low-light mortality and lower growth rates are common for juvenile trees reflected lower shade tolerance [69]. These results support the view that shade-tolerance involves a trade-off between high light growth and low-light survivorship [74–76]. Baraloto et al. [77] also found a negative trend existed between growth rate and survival. Pioneer species are thought to either grow well or die [78]. Higher low-light growth is related to shade-tolerance in tree species as higher growth is often linked to higher survival [76, 79]. However, Davies [69] suggested that this advantage does not hold except in very low light levels where growth of less shade-tolerant species may be lower than growth of more shade-tolerant species. We found similar result evident in our study as the lower mortality probability was found in shade-tolerant and low wood-density species that grew faster than other species groups in the community.

## Conclusions

Overall we found that tree growth, mortality and farmers’ preferences in harvesting timber in species-rich plantations is influenced by both abiotic and biotic factors including anthropogenic influences. Dynamics of tree growth and mortality in these plantings are approaching what might be expected in a natural forest system being driven by the combination of inter-specific and intra-specific competition in these now complex species-rich forest communities. For plantations greater than 10 years old, stand basal area affected individual tree growth negatively but did not appear to be resulting in mortality due to self-thinning from competition when the canopy was closed in these plantings. The higher mortality of shade-intolerant species appears to be the result of light competition, while more harvesting of shade-intolerant species was due to timber demands by farmers. In the early years, shade-intolerant species were preferred to be harvested most likely for local community needs, e.g. house building, fuel wood etc. Our study confirms that generalizable species traits like shade tolerance are an important factor in selecting species for planting and that selective harvesting or thinning of small and shade-intolerant trees could be practiced for high productive stands at age 10+ years may be beneficial in reducing both inter- and intra-specific competition, thus enhancing growth of residual individuals in mixed species stands. Such, the results could be applied in designing a diverse planting; then growth and dynamics of plantings could be predicted based on selected species traits and site characteristics.

## Supporting Information

S1 Fig Tree diameter predicting growth rate of individuals of some common species in the Rainforestation plantings. (DOCX)

S1 File Data of the common species in the Rainforestation plantations. (XLSX)

S1 Table Summary of the common species in the Rainforestation plantations. (DOCX)

S2 Table Variable inflation factor (VIF) of explanatory variables used in modelling. (DOCX)

S3 Table Best candidate models selected from the LMEM models examining tree growth in the community of 32 common species. (DOCX)

S4 Table Best candidate models selected from the LMEM models examining tree status in the community of 32 common species. (DOCX)
