# Supplementary material for: Drivers of Tree Growth, Mortality and Harvest Preferences in Species-Rich Plantations for Smallholders and Communities in the Tropics
Source: PLoS One. 2016 Oct 20;11(10):e0164957. doi: 10.1371/journal.pone.0164957 (PMC5072547; doi:10.1371/journal.pone.0164957)
Supplement: S1 Fig — (DOCX) [file pone.0164957.s001.docx]

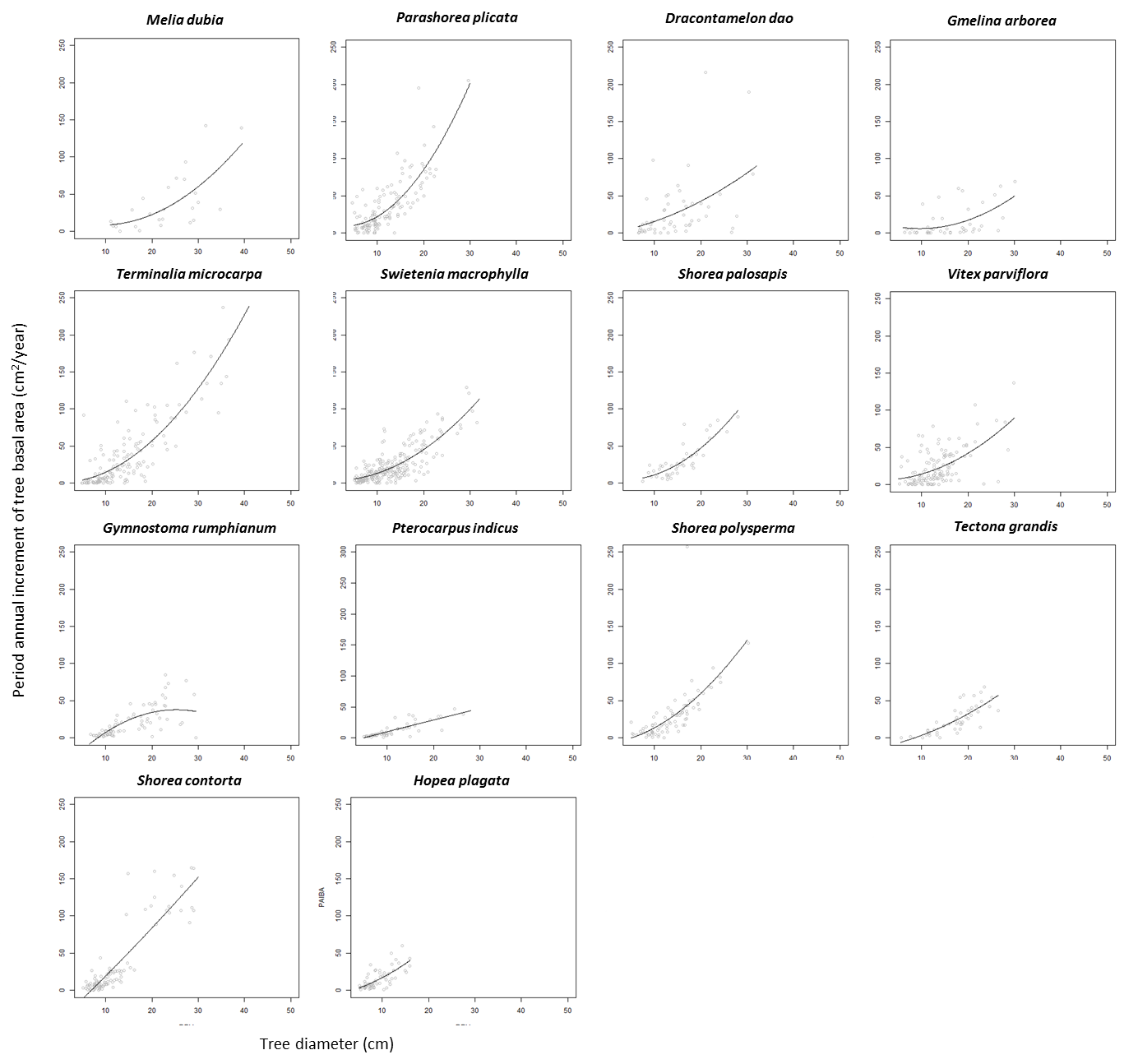


**S5 Fig. Tree diameter predicting growth rate of individuals of some common species in the Rainforestation plantings**
