# Supplementary material for: Drivers of Tree Growth, Mortality and Harvest Preferences in Species-Rich Plantations for Smallholders and Communities in the Tropics
Source: PLoS One. 2016 Oct 20;11(10):e0164957. doi: 10.1371/journal.pone.0164957 (PMC5072547; doi:10.1371/journal.pone.0164957)
Supplement: S1 Table — (DOCX) [file pone.0164957.s003.docx]

**S1 Table. Summary of the common species in the Rainforestation plantations**

| ID | Species | Origin | Shade tolerance |  | DBH±sd (cm) | PAIBA ± sd (cm^2^/year) | Total trees | Dead trees | Harvested trees |
| --- | --- | --- | --- | --- | --- | --- | --- | --- | --- |
|  | *Agathis philippinensis* | Native | Tolerant |  | 12.2±4.2 | 16.9±13.7 | 19 | 1 | 1 |
|  | *Artocarpus blancoi* | Native | Intolerant |  | 12.6±6.4 | 16.1±14.7 | 11 | 1 | 3 |
|  | *Dipterocarpus kunstleri* | Native | Tolerant |  | 11.8±4.7 | 18.4±14.8 | 7 | 0 | 0 |
|  | *Melia dubia* | Exotic | Intolerant |  | 22.1±7.8 | 40.4±35.3 | 24 | 2 | 17 |
|  | *Parashorea plicata* | Native | Tolerant |  | 11.8±4.8 | 25.3±29.9 | 67 | 0 | 0 |
|  | *Calophyllum lancifolium* | Native | Intolerant |  | 9.3±2.4 | 9.7±7.6 | 16 | 0 | 1 |
|  | *Theobroma cacao* | Exotic | Tolerant |  | 10.3±2.4 | 10.3±3.7 | 6 | 0 | 1 |
|  | *Dracontamelon dao* | Native | Intolerant |  | 14.0±6.3 | 21.0±28.5 | 31 | 0 | 4 |
|  | *Durio zibethinus* | Exotic | Tolerant |  | 9.6±2.7 | 10.5±7.4 | 9 | 0 | 0 |
|  | *Gmelina arborea* | Exotic | Intolerant |  | 17.2±9.4 | 40.2±46.5 | 55 | 3 | 31 |
|  | *Myrica javanica* | Native | Intolerant |  | 11.6±2.7 | 15.8±15.1 | 6 | 1 | 1 |
|  | *Leucaena leucocephala* | Exotic | Intolerant |  | 32.3±7.8 | 94.1±56.2 | 9 | 0 | 6 |
|  | *Terminalia microcarpa* | Native | Intolerant |  | 14.6±7.5 | 31.2±41.6 | 89 | 2 | 23 |
|  | *Toona ciliate* | Native | Intolerant |  | 10.2±2.5 | 9.9±7.1 | 5 | 0 | 0 |
|  | *Swietenia macrophylla* | Exotic | Intolerant |  | 13.0±5.7 | 20.1±20.8 | 155 | 1 | 40 |
|  | *Podocarpus rumphii* | Native | Intolerant |  | 6.0±1.3 | 2.5±1.4 | 6 | 0 | 0 |
|  | *Artocarpus odoratissimus* | Exotic | Tolerant |  | 14.8±8.2 | 25.9±24.8 | 7 | 0 | 0 |
|  | *Shorea palosapis* | Native | Tolerant |  | 15.6±4.6 | 28.9±18.6 | 22 | 0 | 0 |
|  | *Vitex parviflora* | Native | Intolerant |  | 13.2±4.9 | 20.0±20.3 | 74 | 2 | 12 |
|  | *Gymnostoma rumphianum* | Exotic | Intolerant |  | 15.4±5.8 | 21.6±17.8 | 63 | 3 | 26 |
|  | *Artocarpus heterophyllus* | Exotic | Intolerant |  | 13.1±3.2 | 11.9±8.9 | 12 | 1 | 6 |
|  | *Pterocarpus indicus* | Native | Intolerant |  | 11.2±5.0 | 11.9±11.5 | 32 | 2 | 7 |
|  | *Samanea saman* | Exotic | Intolerant |  | 19.6±13.3 | 42.5±55.5 | 9 | 0 | 5 |
|  | *Nephelium lappaceum* | Exotic | Tolerant |  | 10.9±3.0 | 14.7±10.8 | 10 | 0 | 0 |
|  | *Sandoricum koetjape* | Native | Intolerant |  | 16.6±5.6 | 35.3±45.2 | 13 | 1 | 6 |
|  | *Pterocymbium tinctorium* | Native | Intolerant |  | 19.0±8.9 | 32.1±34.8 | 10 | 0 | 7 |
|  | *Shorea polysperma* | Native | Tolerant |  | 13.8±4.9 | 26.1±21.8 | 41 | 0 | 1 |
|  | *Tectona grandis* | Exotic | Intolerant |  | 16.5±5.3 | 24.3±16.9 | 26 | 1 | 3 |
|  | *Senna siamea* | Exotic | Intolerant |  | 13.3±4.6 | 20.3±25.2 | 7 | 0 | 2 |
|  | *Shorea contorta* | Native | Tolerant |  | 12.0±6.1 | 24.4±35.7 | 41 | 1 | 0 |
|  | *Hopea malibato* | Native | Tolerant |  | 8.0±3.4 | 9.1±7.4 | 17 | 0 | 1 |
|  | *Hopea plagata* | Native | Tolerant |  | 9.2±2.8 | 12.1±10.9 | 34 | 0 | 2 |
