# Supplementary material for: Drivers of Tree Growth, Mortality and Harvest Preferences in Species-Rich Plantations for Smallholders and Communities in the Tropics
Source: PLoS One. 2016 Oct 20;11(10):e0164957. doi: 10.1371/journal.pone.0164957 (PMC5072547; doi:10.1371/journal.pone.0164957)
Supplement: S2 Table — (DOCX) [file pone.0164957.s004.docx]

**S2 Table. Variable inflation factor (VIF) of explanatory variables used in modelling**

|  | Variable | VIF |
| --- | --- | --- |
|  | Species origin | 1.52 |
|  | Species shade-tolerance | 1.37 |
|  | Species richness | 19.42 |
|  | Species effectiveness | 18.57 |
|  | Tree age | 1.51 |
|  | Tree diameter | 1.22 |
|  | Tree density | 1.83 |
|  | Stand basal area | 1.82 |
|  | Location | 1.17 |
|  | Slope | 1.52 |
|  | Soil type | 1.79 |
