# Supplementary material for: Drivers of Tree Growth, Mortality and Harvest Preferences in Species-Rich Plantations for Smallholders and Communities in the Tropics
Source: PLoS One. 2016 Oct 20;11(10):e0164957. doi: 10.1371/journal.pone.0164957 (PMC5072547; doi:10.1371/journal.pone.0164957)
Supplement: S3 Table — (DOCX) [file pone.0164957.s005.docx]

**S3 Table. Best candidate models selected from the LMEM models examining tree growth in the community of 32 common species**

| Model | Best candidate models | df | AICc | ΔAICc |
| --- | --- | --- | --- | --- |
| LMEM1 | Origin + Shade + Origin*Shade | 7 | 14842.05 | 0.00 |
| LMEM2 | DBH + eH + Origin + Shade + Stand BA + Origin*Shade | 10 | 13808.82 | 0.00 |
|  | DBH + Origin + Shade + Stand BA + Origin*Shade | 9 | 13809.14 | 0.33 |
| LMEM3 | DBH + eH + Location + Origin + Shade + Slope + Soil type + Stand BA + Origin*Shade | 13 | 13797.48 | 0.00 |
|  | DBH + Location + Origin + Shade + Slope + Soil type + Stand BA + Origin*Shade | 12 | 13797.78 | 0.30 |
| Final model | DBH + eH + Location + Origin + Shade + Slope + Soil type + Stand BA + Origin*Shade | 13 | 13797.48 |  |


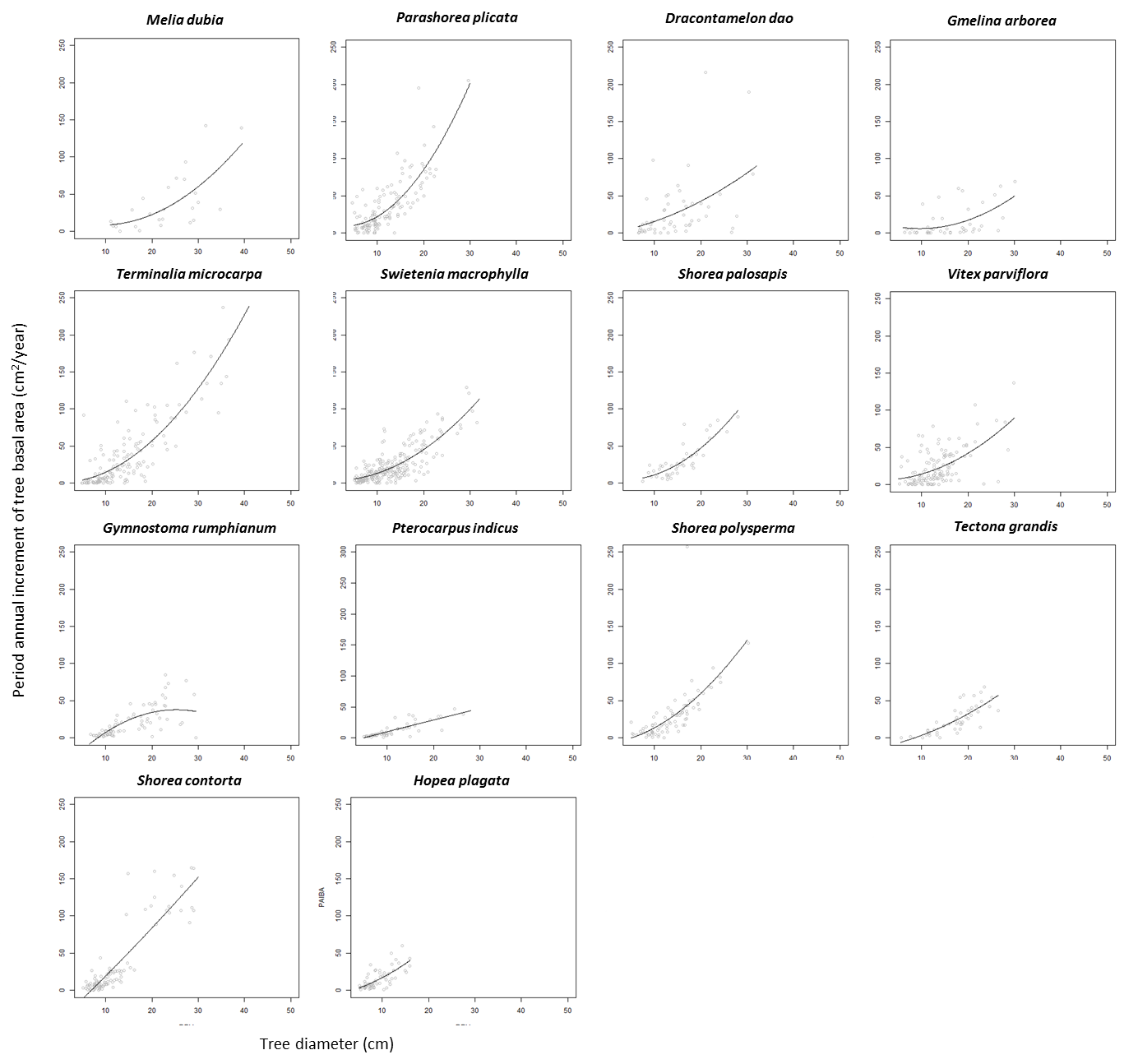


**S5 Fig. Tree diameter predicting growth rate of individuals of some common species in the Rainforestation plantings**
