# Supplementary material for: Drivers of Tree Growth, Mortality and Harvest Preferences in Species-Rich Plantations for Smallholders and Communities in the Tropics
Source: PLoS One. 2016 Oct 20;11(10):e0164957. doi: 10.1371/journal.pone.0164957 (PMC5072547; doi:10.1371/journal.pone.0164957)
Supplement: S4 Table — (DOCX) [file pone.0164957.s006.docx]

**S4 Table. Best candidate models selected from the LMEM models examining tree status in the community of 32 common species**

| Model | Best candidate models | df | AICc | ΔAICc |
| --- | --- | --- | --- | --- |
| LMEM1 | Shade | 5 | 2862.23 | 0.00 |
| LMEM2 | DBH + Shade | 6 | 2858.32 | 0.00 |
| LMEM3 | DBH + Shade | 6 | 2858.32 | 0.00 |
| Final model | DBH + Shade | 6 | 2858.32 |  |
